# Manus Track Preservation Bias as a Key Factor for Assessing Trackmaker Identity and Quadrupedalism in Basal Ornithopods

**DOI:** 10.1371/journal.pone.0054177

**Published:** 2013-01-22

**Authors:** Diego Castanera, Bernat Vila, Novella L. Razzolini, Peter L. Falkingham, José I. Canudo, Phillip L. Manning, Àngel Galobart

**Affiliations:** 1 Grupo Aragosaurus-IUCA, Paleontología, Facultad de Ciencias, Universidad de Zaragoza, Zaragoza, Spain; 2 Institut Català de Paleontologia Miquel Crusafont, Sabadell, Barcelona, Spain; 3 Department of Comparative Biomedical Sciences, Structure & Motion Laboratory, Royal Veterinary College, London, United Kingdom; 4 Department of Ecology and Evolutionary Biology, Division of Biology and Medicine, Brown University, Providence, Rhode Island, United States of America; 5 School of Earth, Atmospheric and Environmental Sciences, University of Manchester, Manchester, United Kingdom; 6 Department of Earth and Environmental Science, University of Pennsylvania, Philadelphia, Pennsylvania, United States of America; Ludwig-Maximilians-Universität München, Germany

## Abstract

**Background:**

The Las Cerradicas site (Tithonian–Berriasian), Teruel, Spain, preserves at least seventeen dinosaur trackways, some of them formerly attributed to quadrupedal ornithopods, sauropods and theropods. The exposure of new track evidence allows a more detailed interpretation of the controversial tridactyl trackways as well as the modes of locomotion and taxonomic affinities of the trackmakers.

**Methodology/Principal Findings:**

Detailed stratigraphic analysis reveals four different levels where footprints have been preserved in different modes. Within the tridactyl trackways, manus tracks are mainly present in a specific horizon relative to surface tracks. The presence of manus tracks is interpreted as evidence of an ornithopod trackmaker. Cross-sections produced from photogrammetric digital models show different depths of the pes and manus, suggesting covariance in loading between the forelimbs and the hindlimbs.

**Conclusions/Significance:**

Several features (digital pads, length/width ratio, claw marks) of some ornithopod pes tracks from Las Cerradicas are reminiscent of theropod pedal morphology. This morphological convergence, combined with the shallow nature of the manus tracks, which reduces preservation potential, opens a new window into the interpretation of these tridactyl tracks. Thus, trackmaker assignations during the Jurassic–Cretaceous interval of purported theropod trackways may potentially represent ornithopods. Moreover, the Las Cerradicas trackways are further evidence for quadrupedalism among some basal small- to medium-sized ornithopods from this time interval.

## Introduction

Morphological similarities between ornithopod and theropod dinosaur tracks have often led to the interchange of taxonomic affinities. The identity of small- to medium-sized gracile tridactyl trackmakers is often disputed [1]–[8]. Traditionally, some of the criteria used to attempt to distinguish between ornithopod and theropod tracks have included: footprint proportions (length/width ratio), the shape of the digits (V-shaped versus U-shaped), the digital extremities (claw marks), the length of digit III, the width and curvature of the digits, the orientation of the hallux, divarication of digits II–IV, interdigital angles, the rear margin of the footprint, presence of interdigital webbing, footprint rotation, and the presence of drag-marks (see [6] and [9] for discussion). Several authors have previously attempted to use multivariate analysis to discriminate between theropod and ornithopod tracks [10], [11], [12], but ultimately these analyses are based upon variations of the above parameters, all of which may be subject to considerable variation depending on factors implicit in both track formation and preservation [8], [13], [14], [15], [16], [17], [18]. Nonetheless, the occurrence of manus tracks associated with pes tracks and forming a well-defined trackway pattern seems to be unequivocal evidence in favour of an ornithopod instead of a theropod affinity [19], [20], [21], [22], [23].

Leonardi [24] suggested that many bipedal trackways of ornithopod dinosaurs might belong to facultative or obligate quadrupedal trackmakers. He suspected that all large ornithopods were quadrupedal, at least facultatively, and that they would produce very shallow and small manus tracks that would be highly susceptible to preservation bias, weathering and erosion after exposure, or oversight. This phenomenon was demonstrated experimentally in both sauropods and ornithopods by Falkingham et al [25], [26], who showed that variations in centre of mass position, combined with differential foot surface areas between manus and pes could, under specific substrate conditions, result in extremely shallow manus impressions from obligate quadrupeds, even when the substrate allowed pes tracks of considerable depth.

Well-preserved trackways of quadrupedal ornithopods (with complete pes-manus sequences) have been reported in several localities throughout the Cretaceous, being more abundant in post-Berriasian ([19], [20], [21], [22] and references therein). During the last decade, sites from the latest Jurassic–earliest Cretaceous (Tithonian–Berriasian) interval have also been reported in Europe that preserve quadrupedal ornithopod trackways [27], [28], [29], [30], [31], [32].

Las Cerradicas (Galve, Teruel, Spain) represents one of the oldest examples of footprint localities with quadrupedal ornithopod trackways [22], [23], [27]. In the initial description of Las Cerradicas, Pérez-Lorente et al. [27] attributed trackway LCR4 to an ornithopod trackmaker and reported three additional tridactyl trackways (LCR1, LCR2 and LCR3) that they tentatively attributed to theropod dinosaurs, though they acknowledged that an ornithopod origin could not be ruled out. Cleaning work carried out in 2007 with the purpose of covering and protecting the site for the IDPI (Dinosaur Ichnites of the Iberian Peninsula) project revealed new tracks and trackways of sauropod affinity (LCR9-LCR14, [33]), small- to medium-sized tridactyl trackmakers (LCR5, LCR6, LCR7, LCR17-LCR19), and more quadrupedal ornithopod dinosaurs (LCR8, LCR15, LCR16, LCR18). In this paper we aim to describe the preservation of the tracks present at Las Cerradicas, and to explore the bias in exposure potential experienced by the shallower manus tracks, compared to the deeper pes impressions. Thus, the quadrupedal ornithopod trackways, the small tridactyl tracks and the previously reported trackways are described here in detail, with the latter reinterpreted in light of the recent discoveries. Moreover, the palaeobiological and palaeoecological implications are discussed.

### Geological setting and track preservation

The dinosaur tracks of Las Cerradicas are located towards the top of the Villar del Arzobispo Formation, which is Tithonian–Berriasian in age. The top of the formation in Galve is probably early Berriasian. In the village of Galve this formation represents transitional deposits from shallow marine platform to terrestrial palaeoenvironments ([27], [33], [34] and references therein).

**Figure 1:**
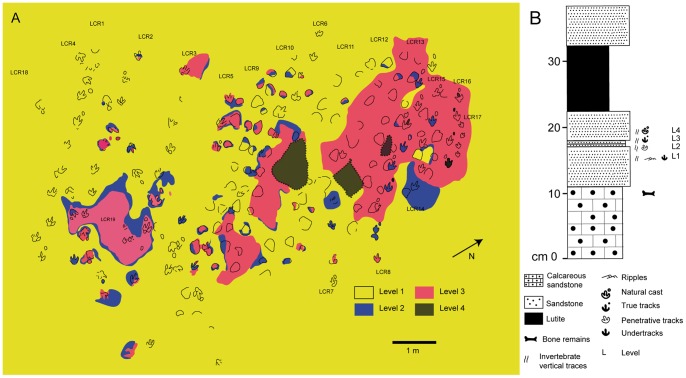
Detailed overview map of the Las Cerradicas site. A) Sketch of the site showing the occurrence of the different levels (modified from [35]). Scale  = 1 m. B) Stratigraphic log of the layers that crop out at the Las Cerradicas site.

**Figure 2:**
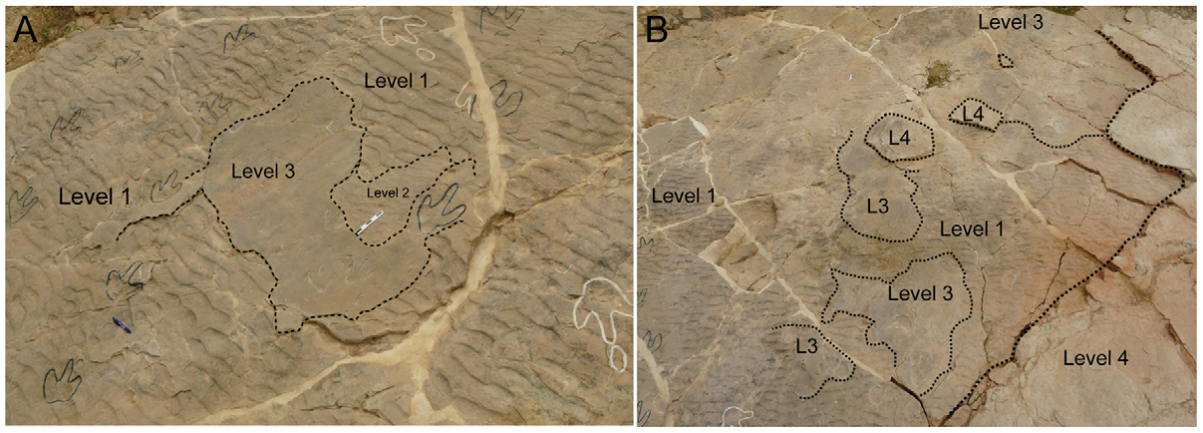
Panoramic pictures of the Las Cerradicas site showing the different levels. A) Picture from the old part of the tracksite showing the levels 1, 2 and 3. Note the “island” just in the middle of the picture. B) Picture from the new part of the tracksite showing the levels 1, 3 and 4.

The site consists of a small outcrop covering ∼80 m^2^, composed of four distinct siliciclastic levels (Figs. 1 and 2). Lithologically, these levels are mainly fine-grained sandstones (in levels 2 and 3 the grain size is smaller than that of levels 1 and 4) that were deposited in a coastal plain setting [27], [33], [34]. The new area cleaned in 2007 (mainly the upper part of Fig. 2B), exposed additional tracks that displayed new preservational types such as natural casts from the true tracks. These new preservation types are studied here for the first time. In the new area (from trackways LCR5 to LCR17) level 4 was excavated, leaving levels 1 and 3 forming the majority of the outcrop (Fig. 2B) as well as level 2 in some minor parts. The new area covers more than half of the surface area of the complete site and allows a better differentiation of the stratigraphic levels and a reinterpretation of the old part where an “island” of levels 2 and 3 is still visible. (Figs. 1, 2A). True tracks and undertracks (transmitted prints) are the most abundant track types, together with natural casts and some penetrative tracks (‘underprints’, *sensu* Marty [18], [36]). The most extensive surface of the site (Figs. 1 and 2) is represented by the lowermost level (upper surface of layer 1, yellow part in Fig. 1, Fig. 2), which is 7 cm thick and characterized by ripple marks. The overlying level (layer 2, blue part in Fig. 1) only crops out in small areas of the site (Figs. 1 and 2) and is only about 1 cm thick. In some parts of the tracksite it is difficult to distinguish this level from the overlying level. In both levels 1 and 2 the tracks are preserved as undertracks (or transmitted prints). The third level (layer 3) is also 1 cm thick and mainly crops out in the eastern part of the site (cleaned in 2007) and in some other isolated small outcrops (Figs. 1 and 2). The tracks are preserved mostly as true tracks or in some cases as penetrative tracks that have penetrated into deeper substrates below layer 3. For this reason we consider layer 3 to be the original palaeosurface upon which at least some of the dinosaurs walked, and in consequence the tracks on this level display the best anatomical details. The uppermost level (layer 4) crops out in the eastern part of the site (Fig. 2B), is about 7–9 cm thick and fills the tracks on top of layer 3, forming natural casts that were recovered during excavation and are provisionally stored at the Museo Paleontológico of the University of Zaragoza. Some tracks from level 3 still preserve part of the infilling of the overlying level 4.

## Materials and Methods

**Figure 3:**
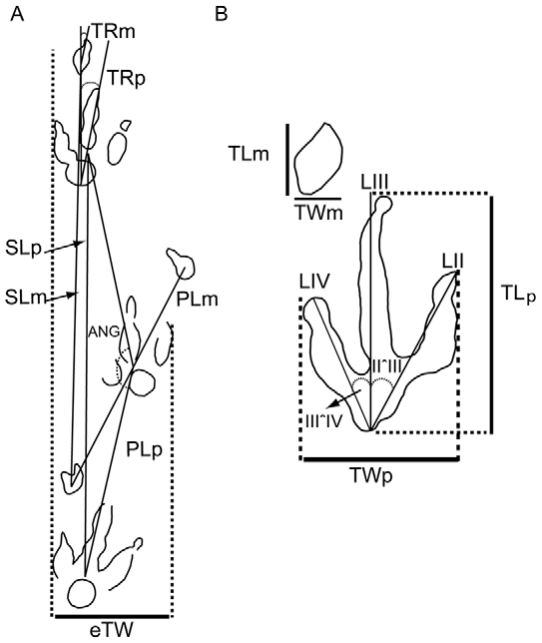
Measurements taken from the tracks. A) Measurements taken for the whole trackway (trackway redrawn and modified from [22]). B) Measurements taken for the individual tracks. Abbreviations: see text in Materials and Methods.

The tracks and trackways were labelled using the procedure adopted by Pérez-Lorente et al. [27] and Castanera et al. [33]. Thus the acronym LCR refers to Las Cerradicas and the number is the position of the trackway in the site. They were numbered from West (LCR1) to East (LCR19) across the outcrop (note that the trackways LCR4 and LCR18 cross the outcrop in a different direction and LCR17–LCR19 represent isolated tracks). Track and trackway parameters were measured directly in the field and from photographs using the software ImageJ, for trackways LCR8, LCR15, and LCR16, and for isolated tracks LCR17-LCR19, which are the best preserved in layer 3 (Appendix S1). The terminology used in this paper mainly follows the works of Thulborn [9] and Marty [36]. Measurements were taken (Fig. 3, Appendix S1) for the footprint length (FL), footprint width (FW), manus–pes distance (Dm–p), length of the digits (LII, LIII, LIV), interdigital angles (II–III, III–IV), pace length (PL), stride length (SL), pace angulation (ANG), footprint rotation (FR), and external trackway width (eTW). The m/p refers to manus and pes, respectively. Speed has been calculated using the Alexandeŕs formula [37] for comparative purposes: h = 4FL; v = 0.25 g^0.5^*SL^1.67^*h^−1.17^.

**Figure 4:**
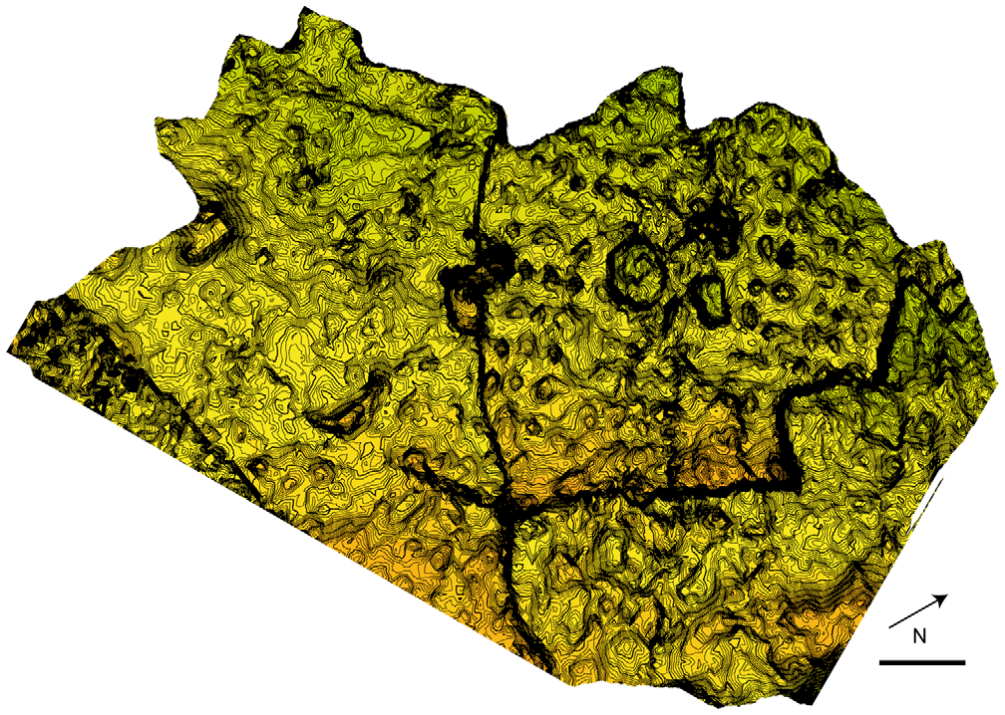
Digital Outcrop Model of the site made with the software Geomagic® Studio 10 and Petrel (**® Schlumberger**)**.** Note that the Model has the same orientation than the sketch of the Figure 1. Scale  = 1 m. The contour-line spacing is 5 mm.

A full digital model of the track-bearing outcrop of Las Cerradicas was made using LiDAR (Light Detection And Range, see [38]). LiDAR data acquisition was accomplished using a RIEGL LMS-Z420i 3D laser scanner. After the LiDAR field survey, a polygonal Digital Outcrop Model (DOM) of the site was constructed from raw point data using Geomagic® Studio 10 (Fig. 4), and analysed in Petrel (® Schlumberger).

The large scale laser scan data was combined with high resolution close-range photogrammetry using the methods described by Falkingham [39] in order to produce higher fidelity models with the purpose of comparing variation in geometry and morphology between tracks. Photogrammetric models were generated for some tridactyl tracks (LCR1.7, LCR3.3 and LCR8.7) with the aim of distinguishing manus impressions, which are not easily discernible using traditional methods (mainly for tracks preserved in the lower most level and in the old part of the tracksite). Photogrammetric models were also imported into Petrel in which vertical cross sections were produced in order to quantify the differential depths of manus and pes tracks.

## Results

### Track and trackway morphology, geometry and preservation variation

Two types of tracks and trackways are exposed in Las Cerradicas site: 1) trackways of tridactyl pes tracks with occasional manus tracks, and 2) trackways with small pes and manus tracks of sauropod affinity. The first group are preserved as undertracks, true tracks and natural casts and include the trackways LCR1 to LCR4 (see Figs. 1; Fig. 2) described by Pérez-Lorente et al., [27], the trackway LCR8 briefly described by Lockley [23], [40] and Lockley et al. [22], the trackways LCR5 to LCR7, LCR15 to LCR18, and a set of smaller isolated tracks (LCR17–LCR19) that have not been previously described. The second group includes trackways LCR9 to LCR14, which were assigned by Castanera et al. [33] to titanosauriform sauropods, and are preserved as undertracks, penetrative tracks, true tracks, and natural casts.

**Figure 5:**
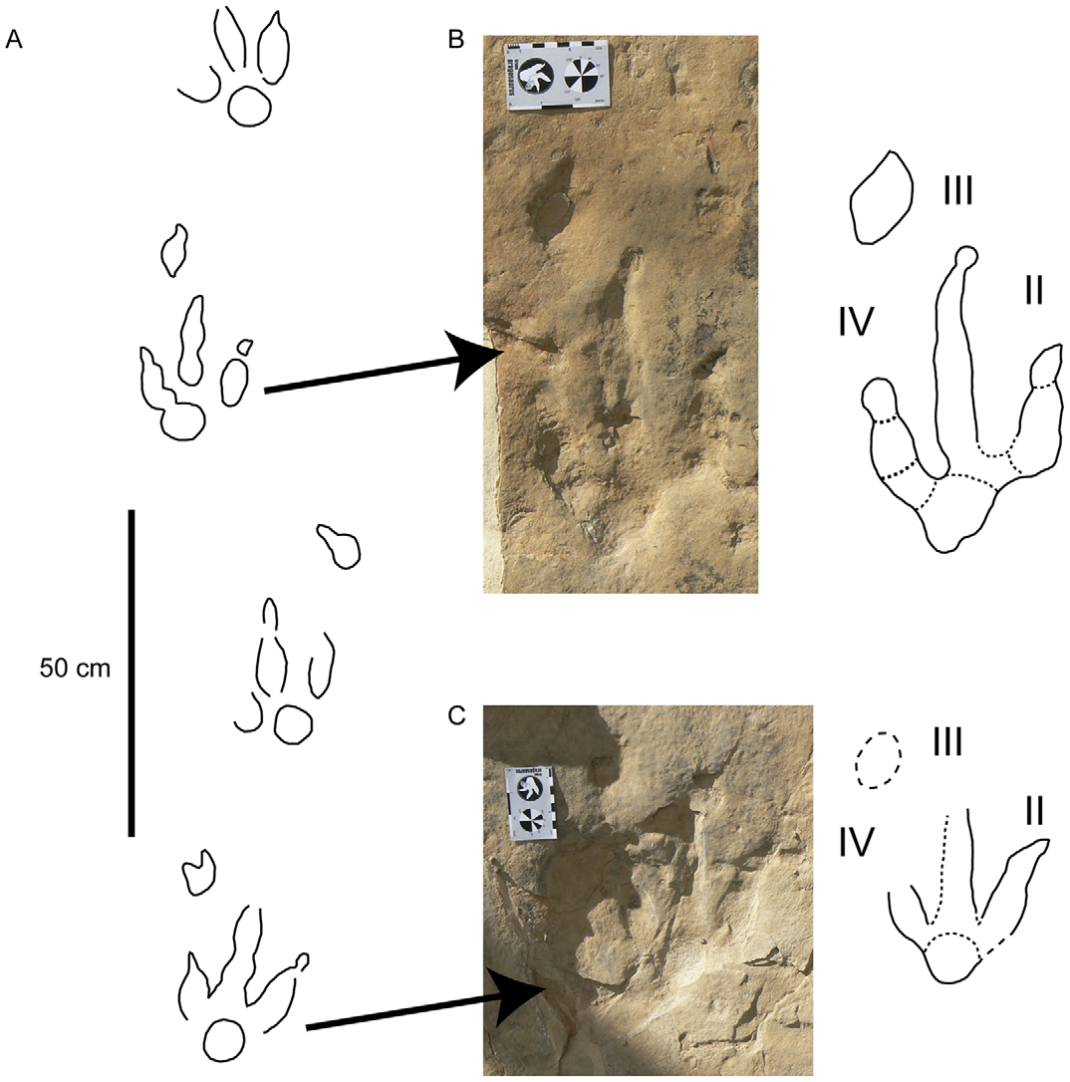
Trackway LCR8 preserved in level 3 as true tracks. A) Sketch of the trackway LCR8 with preserved manus tracks (redrawn from [22]). B) Picture and outline drawing of the pes-manus set LCR8.7. C) Picture and outline drawing of the pes-manus set LCR8.5. Scale (card)  = 8 cm.

**Figure 6:**
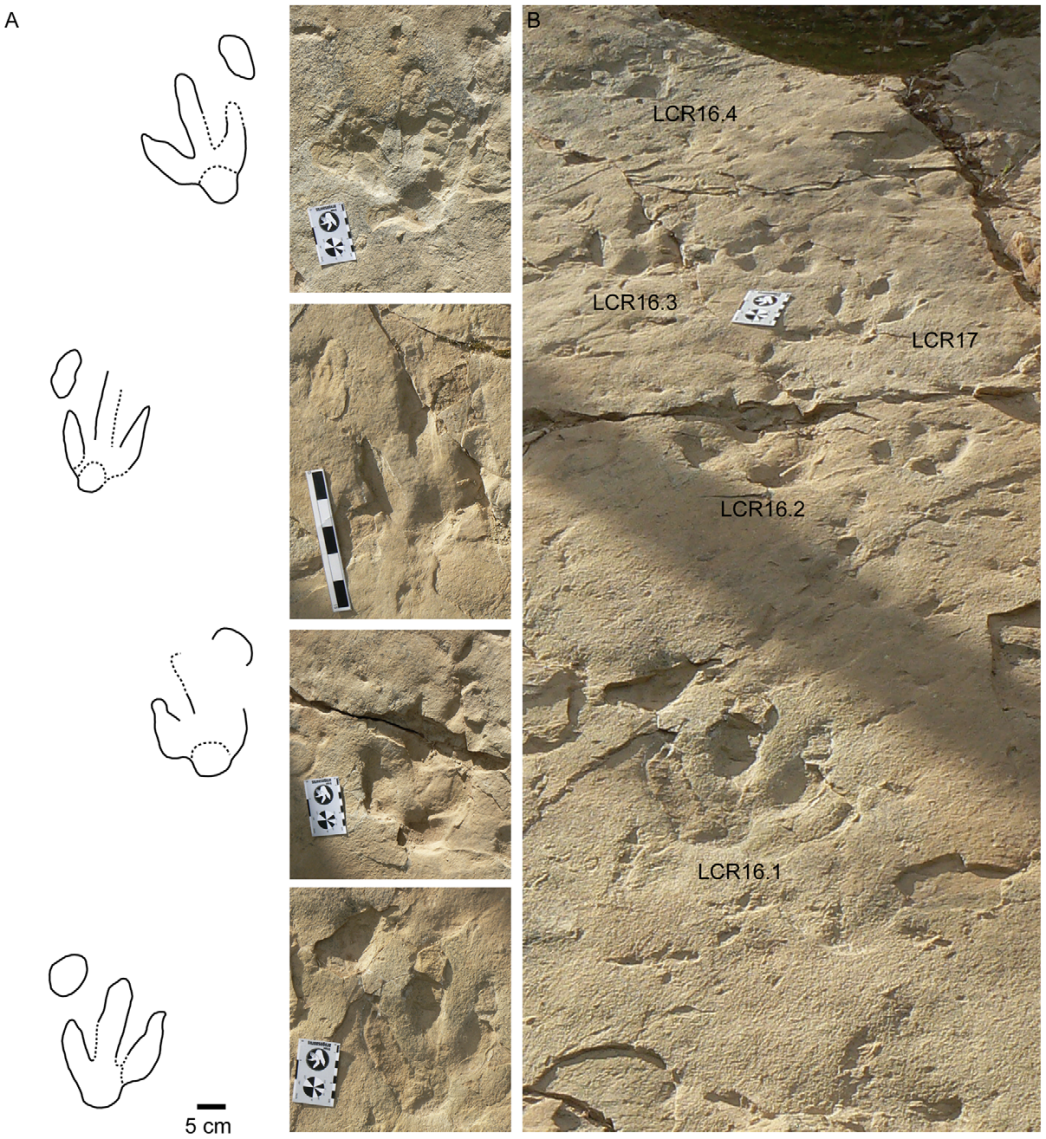
Trackway LCR16 preserved in level 3 as true tracks. A) Sketch and pictures of individual pes-manus sets for the trackway LCR16 with preserved manus tracks. Scale in the pictures 8 cm (card) and 15 (scale bar). B) Picture of the aforementioned trackway and the isolated small tracks LCR17. Note that some of the tracks are infilled by the overlying level 4.

**Figure 7:**
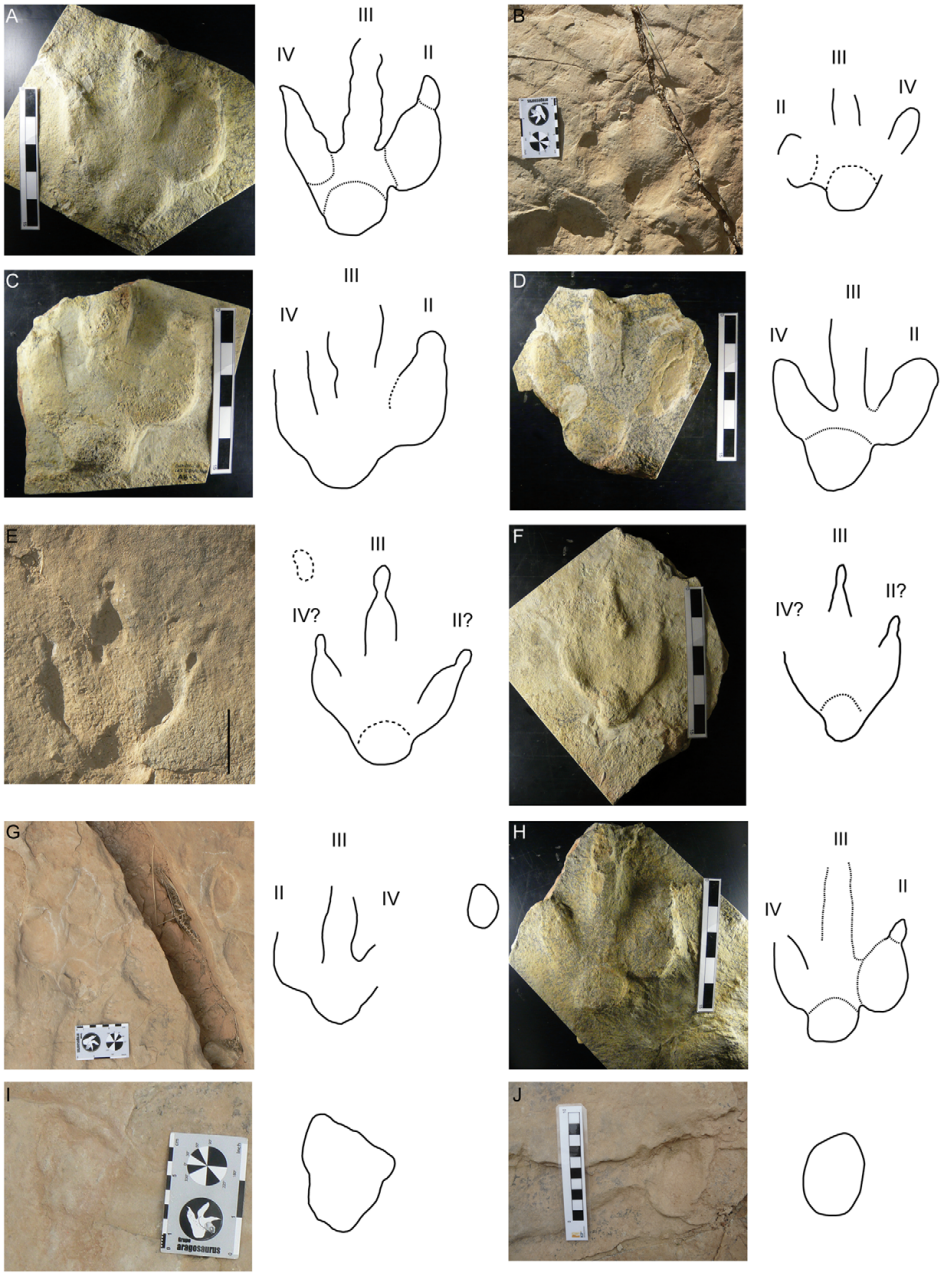
Pictures of some of the best preserved tracks. A) Natural cast of the track LCR15.1p. B) LCR15.4p preserved as a true track. C) Natural cast of the track LCR16.2p. D) Natural cast of the track LCR16.4p. E) LCR17.2pm? preserved as true track. F) Natural cast of the track LCR17.2p. G) LCR18.6p preserved as true track. H) Natural cast of the track LCR18.8?p. I) LCR8.4m preserved as true track with the infilling of the overlying level. J) LCR15.2m preserved as true track. Scale in A, C, D, F, H, J  = 15 cm. Scale in B, G, I  = 8 cm. Scale in E  = 5 cm.

Within the first group (Figs. 5, 6, 7; Appendix S1–S2), the pes tracks are tridactyl, elongate, and longer (23–25 cm for medium-sized and 15 cm for small-sized tracks) than wide (17–18 cm for medium-sized and 11 cm for small-sized tracks). Digit III is considerably longer than digits II and IV and all of them are quite slender and acuminated (V-shaped) at their distal ends. Digit II is slightly wider than digits III and IV. The tip of the digit III is rotated slightly inward in some trackways. The digits have relatively sharp claw traces (when preserved) and discrete phalangeal pads can be recognized in LCR8 (Fig. 5), although phalangeal pads are not well preserved in any natural casts or in other trackways with true tracks (Figs. 6, 7). The tracks usually show symmetry in interdigital angle (II–III = 23–33°, III–IV = 22–30°). Digit II shows a slight indentation on the posterior margin. The posterior margin of the “heel” (distal metatarsal impression) is rounded. The length of the heel impression is approximately one third of the total footprint length.

The manus tracks (when preserved) are oval to sub-oval (Figs. 5, 6, 7) and have the long axis oriented anteromedially with inward rotation. They do not have clearly differentiated digit impressions, although some blunt digit tip traces have been inferred in some tracks [22]. The manus tracks are positioned in front of and slightly outside of the pes tracks, positioned approximately 20 cm more anterior than digits III and IV of the precedent pes track. The pes tracks show inward rotation along the midline of the trackway, and the pace and stride length are from 52–58 cm and 100–110 cm for the manus and pes respectively. The external width of the trackway measured from the pes tracks is 27–28 cm (Appendix S1).

**Figure 8:**
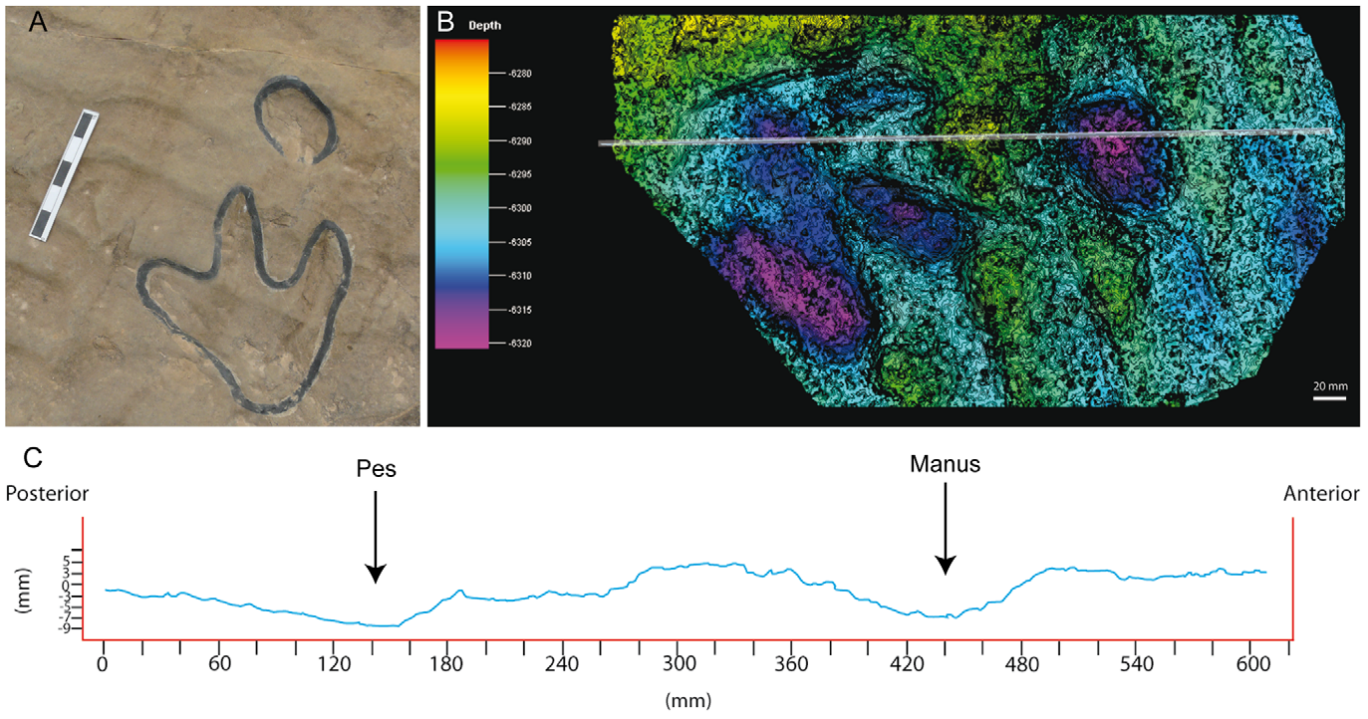
Track LCR1.7 preserved in layer 1 as undertrack. A) Picture of the track. Scale bar  = 15 cm. B) Photogrammetric 3D depth analysis model. The white line represents the longitudinal cross section that crosses the track from the “heel” pad through the digit III to the manus print. The contour-line spacing is 3 mm. The depth units are also mm. C) Cross section profile.

**Figure 9:**
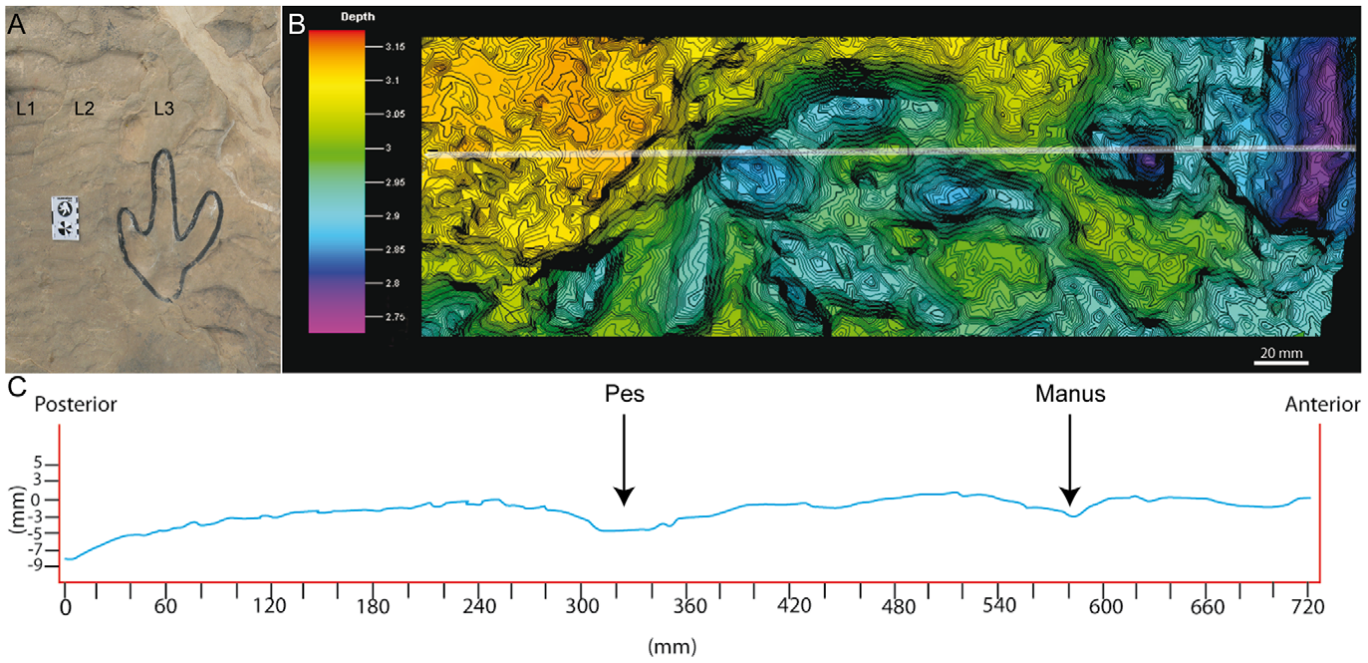
Track LCR3.3 preserved in layer 3 as true track. A) Picture of the track. Note the levels 1 and 2 in the left part of the picture. Scale (card)  = 8 cm. B) Photogrammetric 3D depth analysis model. The white line represents the longitudinal cross section that crosses the track from the “heel” pad through the digit III to the manus print. The contour-line spacing is 3 mm. The depth units are also mm. C) Cross section profile.

**Figure 10:**
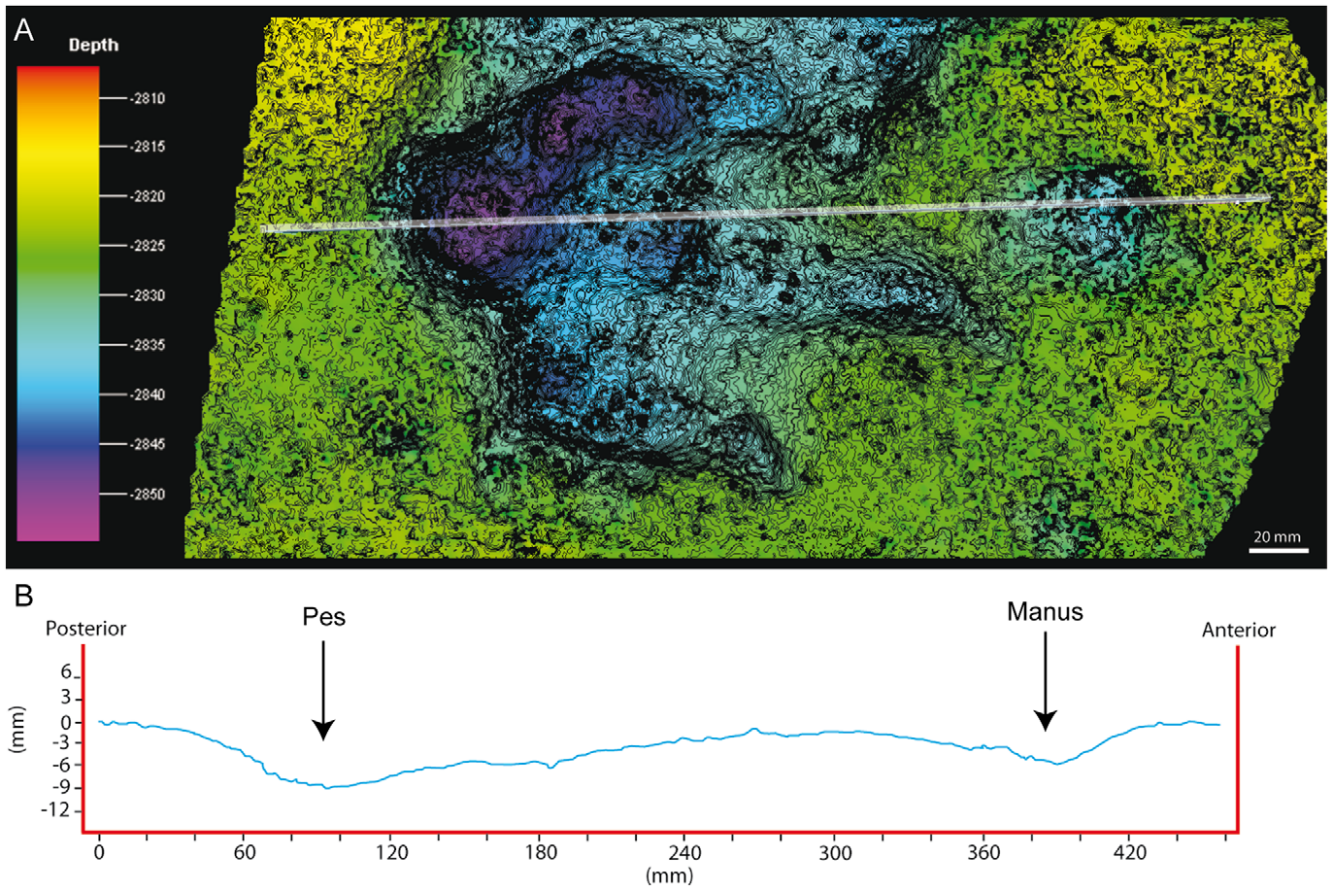
Track LCR8.7 preserved in layer 3 as true track (**compare with the** **figure** **5B**)**.** A) Photogrammetric 3D depth analysis model. The white line represents the longitudinal cross section that crosses the track from the “heel” pad through the digit III to the manus print. The contour-line spacing is 3 mm. The depth units are also mm. B) Cross section profile.

In addition to the larger tracks noted above, there are two sets of smaller tridactyl tracks preserved in layer 3 (LCR17 and LCR19). Four of these (LCR17) are located in the eastern part of the site whereas another set of three tracks (LCR19) is found in the western part (Figs. 1, 2A, 6B, 7E, 7F). Only track LCR17.2 (Fig. 7E) shows an oval depression located in the place where a manus impression would be expected. Otherwise, there is no evidence of associated manus impressions with these tracks. The small tridactyl tracks share a similar morphology to the larger tracks, but the relation between L/W (Appendix S1) is slightly smaller, and they are more symmetrical. There is considerable variation in the general morphology of the tridactyl tracks. This variation depends on the level on which the tracks are exposed. For example, LCR3 and LCR18 are exposed on multiple sedimentary layers throughout individual trackways. Most of the tracks present on layer 1 (mainly tracks of the trackways LCR1 to LCR7, and LCR18) have poorly defined outlines, high divarication angles and there is not a clear distinction between the “heel” and digital areas (Fig. 8); thus, we consider them undertracks (transmitted tracks). The tracks on level 1 (e.g. LCR1.7; Fig. 8A, Appendix S2) do not break or interact with the ripple marks present since these tracks result from load transmission from upper layers and this clearly identifies them as undertracks. Other tracks from the same trackway, as well as some other tracks in nearby trackways LCR2 and LCR3, display similar preservation features and very few of these pes tracks are associated with manus impressions. A cross-section of LCR1.7 reveals (Fig. 8B, 8C) that there is a depth difference between manus and pes tracks. In this case the pes track is 0.9 cm deep whereas manus track is 0.7 cm deep. On level 3 the tridactyl true tracks and their natural casts from level 4 (mainly tracks in the trackways LCR3, LCR8, LCR15 and LCR16, and the isolated tracks LC17–LCR19) display similar morphologies to each other and are therefore considered to belong to a single morphotype. Well-preserved examples of this morphotype are also found in the central parts of levels 2 and 3 that contain tracks LCR3.3 (pes and manus, Fig. 9, Appendix S2). Field observations show that layer 3 is continuous inside the tracks LCR3.3 and LCR8.7 and thus the foot did not penetrate or break the underlying layers (1 and 2), but rather deformed them via transmission of force [8], [25]. Digital cross-sections of LCR3.3 and LCR8.7 (Figs. 9, 10) reveal that the heel impression is the deepest part of the pes track, and that this is always deeper than the manus impression. Thus, there is also a differential depth between manus (depth: 0.3 cm for LCR3.3; 0.6 cm for LCR8.7) and pes (depth: 0.5 cm for LCR3.3; 0.9 cm for LCR8.7) tracks. Comparing the track cross-sections between these three levels (Figs. 8, 9, 10), and assuming an identical trackmaker species, we can observe remarkable differences between them. Only the cross-section from level 3 (Figs. 9C–10B) reveals the real morphology of the pes and the manus, while the cross-section from level 1 (Fig. 8C) is highly influenced by the morphology (ripples) of the substrate, and due to being an undertrack.

## Discussion

**Figure 11:**
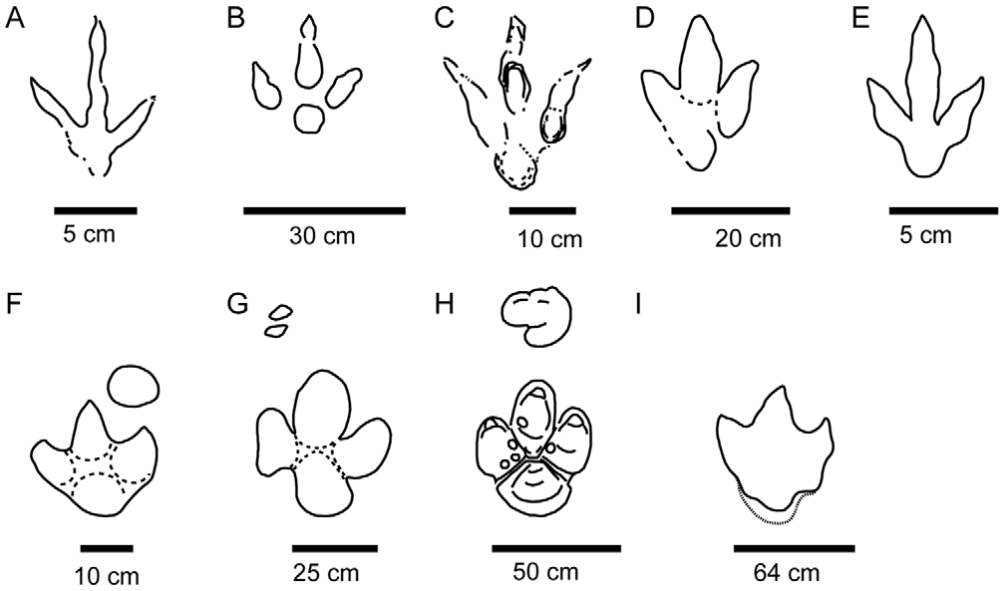
Comparison with some ichnotaxa. A–E) Tridactyl ichnotaxa sharing features with Las Cerradicas tracks: *Toyamasauripus* [2], *Dinehichnus* [5], *Asianopodus* [44], *Therangospodus* [45] or *Kalohipus* [47]. F–I: main “large ornithopod” ichnotaxa from the Early Cretaceous: *Iguanodontipus* [29], [51], *Amblydactilus* [52] or *Caririchnum* [53].

Controversy is not uncommon when identifying tridactyl tracks as being either theropod or ornithischian in the Triassic–Jurassic interval and Early Jurassic (see [41], [42], [43]). In the Late Jurassic–Early Cretaceous it is equally difficult to distinguish between the identification of these track groups. The locality of Las Cerradicas is not an exception to this scenario and thus its tridactyl tracks share similarities with some typical medium-sized bipedal ichnotaxa (Fig. 11) from the Late Jurassic and Early Cretaceous attributed to both theropods and ornithopods, such as *Toyamasauripus* (Fig. 11A, [2]), *Dinehichnus* (Fig. 11B, [5]), *Asianopodus* (Fig. 11C, [44]), *Therangospodus* (Fig. 11D, [45], [46]) and *Kalohipus* (Fig. 11E, [47]). Pérez-Lorente et al. [27] did not completely rule out an ornithopod attribution for the trackways LCR1, LCR2 and LCR3, and Lockley [40] associated the pes tracks of Las Cerradicas with the ichnogenus *Asianopodus* [44] and *Dinehichnus* [5], which are attributed to theropods and dryomorph ornithopods, respectively. Lockley et al. [22] and Lockley [23], [40] considered the trackway LCR8 as ornithopod in origin, and suggested that the tracks “could easily be mistaken for theropod tracks,” being morphologically similar to this group.

The features of the Las Cerradicas tridactyl tracks do not fit well with the characters of large ornithopod tracks (see [48]). Lockley et al. [22] and Lockley [23] suggested that the characters of the Las Cerradicas tridactyl tracks are intermediate between *Dinehichnus* tracks and larger ornithopod tracks such as *Iguanodontipus* and that there is a tendency for smaller ornithopod tracks to be more elongate than large robust forms in the Late Jurassic–Early Cretaceous, but such qualitative measures are difficult to reliably apply to track morphotypes. Both ichnotaxa have been described from the Iberian Peninsula [5], [49], [50], and the latter in deposits similar in age to Las Cerradicas. The similarities between some *Dineichnus* tracks and the small tracks (LCR17-LCR19) from Las Cerradicas (Fig. 7E, 7F) are noteworthy. Las Cerradicas tracks are more gracile than some other typical Early Cretaceous large ornithopod ichnotaxa, such as the aforementioned *Iguanodontipus* (Fig. 11F, 11G, [29], [51]), *Caririchnum* (Fig. 11H, [52]) or *Amblydactilus* (Fig. 11I, [53]). Therefore, it seems that the typical characteristics used to distinguish between large theropods and ornithopods are not well defined when dealing with small-medium tridactyl trackmakers (see discussion in [6], [36]).

The detailed stratigraphic assessment of the Las Cerradicas site, together with the photogrammetric models and cross-sections, is revealed as a pivotal element to decipher the trackmaker identity. The analysis of the site shows that there is a clear preservation bias against manus tracks in all the tridactyl trackways, except isolated tracks LCR17 and LCR19 (Fig. 1). Due to differential erosion, upper layers (layers 2 and 3) have disappeared from half of the outcrop. In the areas where layer 1 is exposed, the tracks are preserved as undertracks from the trampling on overlying level 3 and hence do not preserve much anatomical detail (Fig. 8). Therefore, tracks on level 1 are less detailed versions of the tracks on level 3. Consequently, track outlines are poorly defined and the occurrence of manus tracks is rare. Contrary to the undertracks on level 1, level 3 displays true tracks with substantive anatomical details such as claw marks, or in some cases digital pads impressions. While some of these characters are typical of theropod trackmakers, the presence and the oval morphology of manus tracks, on the other hand, supports an ornithopod origin. Furthermore, the inward rotation of pes tracks, the round heel pad and relatively short steps are typical features of ornithopod rather than theropod trackmakers [19], [20], [21], [22], [23], [40].

The lack of manus tracks in layer 1 may be explained in terms of differential loading (Figs. 8, 9, 10) and preservation relative to the pes of the trackmaker [25], [26], [54]. The differential depths of pes and manus tracks (Figs. 9 and 10) suggest a more caudal centre of mass, producing a greater relative loading under the hind than the forelimb. The differences in depth would be transmitted to underlying levels; thus, we suggest that the hind foot load would be transmitted deeper from layer 3 to layer 1 whereas that of the forelimb would only reach layers 2 and 3 [26]. This provides an alternate explanation to the proposed presence of different dinosaur trackmakers (i.e. theropods versus ornithopods; cf. Pérez-Lorente et al. [27]). Given that the tracks present on layer 1 are interpreted here as undertracks transmitted from layer 3 (at or close to the tracking surface), we consider quadrupedal ornithopods, rather than bipedal theropods to be the producers of all of the tridactyl trackways. Thus, the preservation bias from Las Cerradicas supports the ideas proposed by Lockley [55] and Diedrich [29] that the lack of manus prints is strongly related to substrate deformation and preservation.

Regarding the trackmaker identity within Ornithopoda, Pérez-Lorente et al. [27] suggested that the pes length of the tracks from the trackway LCR4 could fit with the size of trackmakers such as *Camptosaurus dispar* or *Iguanodon atherfieldensis* (now *Mantellisaurus atherfieldensis*). Nonetheless, the pedes of these ornithopods (see [56], [57]) are too robust, and probably larger, to have produced the features of Las Cerradicas tracks, which show gracile digits and sharp claw marks. Ornithopod remains are common in the Early Cretaceous of the Iberian Peninsula [58], [59], [60] but are scarce at the Jurassic–Cretaceous transition (Tithonian–Berriasian). The ornithopod skeletal record is poorly represented in the Villar del Arzobispo Formation (Tithonian–Berriasian) compared with other dinosaurs, especially sauropods [61], [62]. To date, there is only an isolated tooth assigned to *Valdosaurus* [63], a dryosaurid that was relatively common in the Early Cretaceous of Europe [64], and cranial and postcranial material from a basal ankylopollexian found in a new Tithonian–Berriasian locality [65]. Both groups, dryosaurids and basal members of Ankyllopollexia, are the most abundant ornithopods in the Late Jurassic of the Iberian Peninsula [58], [59] and as yet the unique from the Tithonian-Berriasian. Thus, the most probable candidates for Las Cerradicas trackmakers would be quadrupedal representatives of basal ornithopods (possibly dryosaurids or basal members of Ankyllopollexia).

### Palaeobiological and Palaeoecological implications

The preservation bias observed in the ornithopod manus tracks at Las Cerradicas has two main implications for the interpretation of the palaeobiology and palaeoecology of the site. The first relates to the locomotion of basal ornithopods (quadrupedalism) and the second to the possible gregarious behaviour represented.

Considering ornithopod locomotion, trackways of small–medium sized quadrupedal ornithopods are interesting because the majority of such trackways are much larger and often attributed to iguanodontids and hadrosaurs [19], [20], [21], [29]. The derived ornithopods such as hadrosaurs are considered predominantly quadrupedal while more basal forms such as “hypsilophodontids” are considered bipedal [66], [67]. Between these basal and derived groups of ornithopods locomotion is not well understood [66], [67] and quadrupedality inferred from trackways is scarcely documented for medium-sized ornithopods in the Late Jurassic–Early Cretaceous worldwide, except in Europe [27], [28], [29], [30], [31], [32]. In fact, some authors [68] have proposed that there is a relationship between ontogenetic state and bipedalism/quadrupedalism in some dryosaurids. Heinrich et al. [68] proposed that hatchlings of some dryosaurids would have been obligate quadrupeds while the adults were bipedal. This case is the opposite of what is reflected in the tracks from Las Cerradicas, where in layer 3 among the true tracks the small tracks apparently (LCR17.2 could have preserved the manus, Fig. 7E) did not preserve manus tracks while the medium-sized tracks did. The scarce occurrence of manus tracks among the small tracks could indicate ontogenetic change in the relative loading of the manus and pes, with adults placing more weight on the fore-limbs, or, perhaps more likely, it could simply be a function of the juvenile manus not producing enough pressure to exceed the plastic limit of the substrate. Thus, the track record from Las Cerradicas is significant since it probably represents evidence of quadrupedalism in basal and rather small ornithopods, an observation that has not yet been reported from osteological studies [67]. Nonetheless, we cannot state whether this evidence of quadrupedalism would be indicative of obligate quadrupedalism or an adaptation for concrete surfaces due to substrate properties (facultative quadrupedalism).

**Figure 12:**
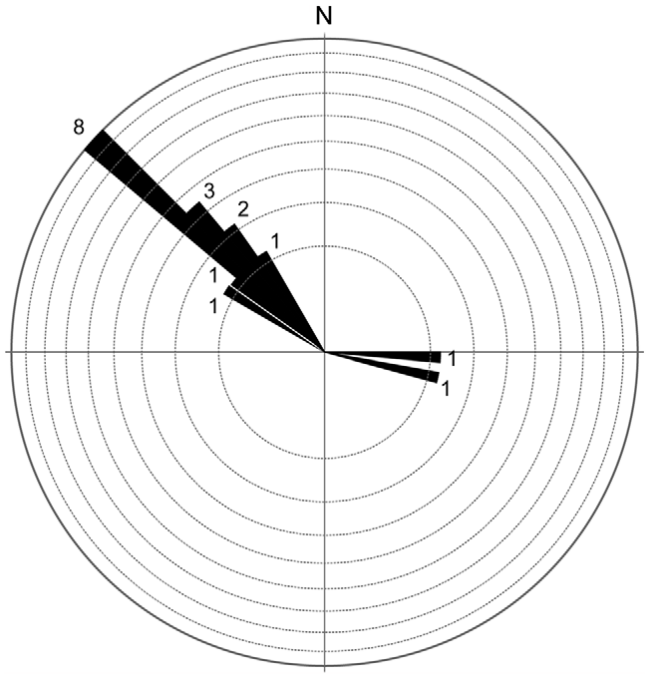
Rose plot showing the orientation of the ornithopod and sauropod trackways from Las Cerradicas site. The orientation unit in the rose diagrams is 5° and the circular lines correspond to one unit (1 trackway).

The gregarious behaviour of small bipedal, tridactyl dinosaurs has been proposed in different localities through geological time ([69] and references therein). In Las Cerradicas, the trackways from the old part of the site (LCR1, LCR2 and LCR3) have some of the typical features (parallel trackways, same morphotype, speed values, close intertrackway spacing, pace rhythm; see methodology in [70]) to suggest that they were possibly moving together. Pérez-Lorente et al. [27] also suggested that these trackways were “moving around in a group”. The new trackways (LCR5, LCR6, LCR7, LCR8, LCR15, LCR16) display features that also suggest possible gregarious behaviour (Figs. 1, 12). Nonetheless, there are significant disparities that are worthy of comment. There are slight variations in the orientation of some trackways (LCR5, LCR6, LCR7), which are poorly preserved. Among the best preserved (LCR8, LCR15, LC16) and those located in the old part of the site (LCR1, LCR2, LC3) the orientation is similar (Figs. 1, 12). The speed values (about 3 Km/h), the close intertrackway spacing and the comparable pace rhythm suggest that LCR1, LCR2, LCR3 and LCR8, LCR15, LCR16 may have been walking together. However, the intertrackway spacing and the pace rhythm of the intermediate trackways (LCR5, LCR6 and LCR7) is not clear enough to suggest that these animals were walking contemporaneously. The scarce number of small tracks and the fact that they apparently are not displayed in trackways prevents their assignment to juvenile animals of the same ichnospecies, though this remains a possibility.

**Figure 13:**
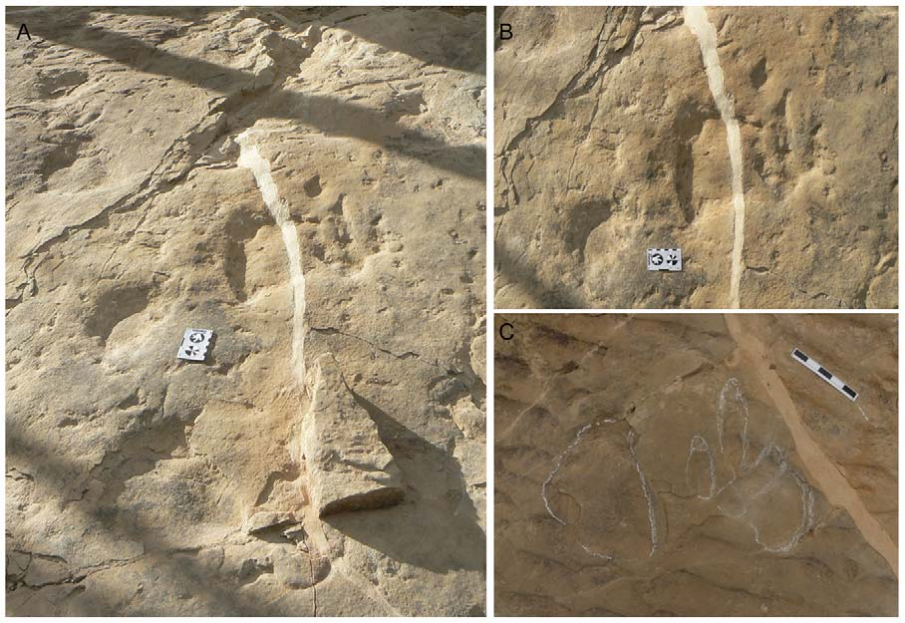
Overprinting relationships between sauropods and ornithopods. A) LCR8.7p overprinting LCR13.6p. B) Detailed of LCR8.7p- LCR13.6p C) LCR6.2p overprinting LCR9.3m.

Furthermore, another interesting feature of the site is that the majority of the trackways (except LCR4 and LCR18) are parallel to those made by sauropod trackmakers. This is worth underscoring since there are only a few sites reported in the literature that record both ornithopod and sauropod trackways on a single surface [36], [70], [71], [72]. At Las Cerradicas the ornithopod trackways LCR6 and LCR8 overprint the sauropod trackways LCR9 and LCR13, respectively (Figs. 1 and 13), so these trackways seem to have been made subsequently. Within the other tracks there are no visible overprinting relationships. We do not have enough data to propose that the ornithopods travelled together with the small sauropods. In fact, it could also be that a similar pathway implied by the local topography was taken independently by two groups of herbivorous dinosaurs, but separated by an unknown time period.

## Conclusions

The Las Cerradicas tracksite provides unique data for the study of basal ornithopod pes and manus track morphology in the Jurassic–Cretaceous interval. The morphological convergence of the pes prints with those of theropod origin makes the task of differentiating between these groups fraught with difficulty, and reliant upon the presence of manus impressions. The shallow and poorly-preserved manus tracks are highly susceptible to preservation bias (being preserved only as shallow marks, and on fewer surfaces than the deeper pes tracks). As such an ornithopod origin for all the tridactyl trackways is possible, and we consider this to be more parsimonious than invoking additional trackmakers. The erosion of just a centimetre of rock is enough to entirely obliterate the manus impressions, whilst still leaving the pes tracks, which demonstrates that the trackmaker identification of small–medium sized trackmakers should be treated with caution. If these tracks were produced by a quadrupedal ornithopod, this suggests that at least some basal ornithopods (maybe dryosaurids or basal members of Ankyllopollexia) were at least facultatively quadrupedal. The parallel orientation of the trackways and similar state of substrate deformation, possibly indicate gregarious behaviour among these basal ornithopod trackmakers at the time of track formation. However, time resolution for the relative formation of tracks in vertebrate ichnoassemblages will remain a point of contention. Las Cerradicas does however represent a rare example where both ornithopod and sauropod tracks co-occur on a single level (ichnoassemblage).

## Supporting Information

Appendix S1 **Table with the measurements taken in the ornithopod trackways at Las Cerradicas tracksite.** Abbreviations: see text in Materials and Methods.
(XLS)

Appendix S2 **Photogrammetric models of the pes-manus sets LCR1.7, LCR3.3, LCR8.7 from Las Cerradicas tracksite. Scale bar: 15 cm.**
(RAR)
