# Correction: Manus Track Preservation Bias as a Key Factor for Assessing Trackmaker Identity and Quadrupedalism in Basal Ornithopods

**DOI:** 10.1371/annotation/e0dba720-9eda-4457-ae59-24d9d96eb8c8

**Published:** 2013-09-20

**Authors:** Diego Castanera, Bernat Vila, Novella L. Razzolini, Peter L. Falkingham, José I. Canudo, Phillip L. Manning, Àngel Galobart

Multiple funding organizations and grants were incorrectly omitted from the Funding Statement. The Funding Statement should read: "This paper forms part of the projects CGL2010-16447 and CGL2011-30069-C02-01, subsidized by the Ministry of Economics and Competition, the European Regional Development Fund and the Government of Aragón ("Grupos Consolidados" and "Dirección General de Patrimonio Cultural"). DC is the beneficiary of a grant from the Ministry of Education (AP2008-01340). BV acknowledges support from the Ministry of Science and Innovation (Subprograma Juan de la Cierva (MICINN-JDC) 2011). PLF was supported by a Marie Curie International Outgoing Fellowship within the 7th European Framework Programme. Lidar acquisition was funded by Institut Català de Paleontologia (ICP) and the School of Earth, Atmospheric and Environmental Science, University of Manchester. The funders had no role in study design, data collection and analysis, decision to publish, or preparation of the manuscript." 

